# Laser jetting of femto-liter metal droplets for high resolution 3D printed structures

**DOI:** 10.1038/srep17265

**Published:** 2015-11-25

**Authors:** M. Zenou, A. Sa’ar, Z. Kotler

**Affiliations:** 1 Additive Manufacturing Lab, Orbotech Ltd. P.O. Box 215, Yavne 81101, Israel; 2 Racah Institute of Physics and the Harvey M. Kruger Family Center for Nano-science and Nanotechnology, the Hebrew University of Jerusalem, Jerusalem, Israel

## Abstract

Laser induced forward transfer (LIFT) is employed in a special, high accuracy jetting regime, by adequately matching the sub-nanosecond pulse duration to the metal donor layer thickness. Under such conditions, an effective solid nozzle is formed, providing stability and directionality to the femto-liter droplets which are printed from a large gap in excess of 400 μm. We illustrate the wide applicability of this method by printing several 3D metal objects. First, very high aspect ratio (A/R > 20), micron scale, copper pillars in various configuration, upright and arbitrarily bent, then a micron scale 3D object composed of gold and copper. Such a digital printing method could serve the generation of complex, multi-material, micron-scale, 3D materials and novel structures.

Digital printing of metals is probably the single most important element missing from functional 3D printing, a technology that today still relies almost entirely on polymer materials. 3D structures made up of polymers usually lack the required mechanical, electrical and thermal properties for functional structures and devices. Metal deposition by conventional methods, either by evaporation or by electrochemistry, is quite inadequate for fast build-up, multi-layered 3D structures. Currently the main approach for printing metals is based on metal inks, nano or micron scale metal particle ink formulations^1^,^2^,^3^,^4^,^5^,^6^,^7^,^8^,^9^ which can be printed and then sintered to obtain a metallic layer. Typically, such metal inks and pastes were developed for printing methods used in the graphic arts industry, such as screen printing^10^,^11^,^12^, inkjet printing^1^,^2^,^3^,^4^,^5^,^6^, flexography and gravure^13^,^14^,^15^. However, there are several well-known limitations with printing inks. The metals offering is rather limited with the options offered are basically limited to silver, gold or copper. In addition, the printed object geometry is constrained by the liquid wetting properties and the post-printing thermal sintering step even further limits the substrate material choice.

Metal micro-droplets can be printed^16^,^17^,^18^ directly from the bulk solid phase through laser induced forward transfer (LIFT)^19^,^20^ overcoming the limitations associated with printing metal inks. LIFT printing relies on a so-called ‘donor’ that consists of a transparent substrate coated by a thin layer of the print material (typically with a thickness of a few tens of nanometers) (Fig. 1a). A laser pulse focused on the interface between the metal layer and the substrate induces local thermal heating followed by a phase change and high local pressure which drives the jetting of the print material. Recent reports described sub-micron metal droplet jetting^21^,^22^,^23^ using femto-second pulses. Figure 1a schematically describes the mechanism involved in LIFT jetting in the ‘metal pool’ case, where the entire metal layer thickness is melted locally during the laser pulse duration. The (high) thermally induced pressure at the substrate-liquid interface then drives the droplet formation and jetting out of the transient molten liquid layer^24^,^25^. Droplets, which emerge from such a molten metal layer, typically have limited directionality and as a result, the print accuracy is rather low unless the donor is brought into very close proximity, typically a few tens of microns, to the acceptor substrate^16^,^17^,^18^,^19^,^20^,^21^,^22^,^23^,^24^,^25^.

In this work we describe a new jetting mechanism which provides a stable, highly directional jetting of metal droplets from a rather large distance (>400 μm), therefore overcoming the basic limitations of the “melt-pool’ LIFT printing case. This is primarily made possible due to a different jetting mechanism which is effective when using sub-nanosecond pulses and relatively thick metal donor layers (thickness >300 nm). Figure 1b describes this case schematically; it involves the formation of a thermally induced quasi-nozzle that, unlike the cases described previously, provides high directionality to the emerging droplets. Using sub-nanosecond pulses (~0.4 ns), a ~300 nm thick copper layer would still melt all the way to the free surface within the pulse duration. However, for a layer >300 nm thick, the thermal diffusion length within the pulse duration is smaller than the layer thickness (the detailed relationship is described below in relation to Fig. 2). For the molten metal front to reach the free surface through heat diffusion, the pulse energy has to be increased to allow for the excess thermal energy needed for the melt front to still propagate even after the pulse has ceased. The molten material front will indeed reach the free surface by thermal diffusion however, at the same time, a solid wall will form around the central melt region forming an opening (Fig. 2c), the so-called “thermally induced nozzle” (TIN). This solid and circularly symmetric aperture provides high directionality to the molten metal droplet as it is ejected. We demonstrate how this TIN mechanism provides stable jetting with very low angular divergence in the case of copper layers of 500 nm thickness and 400 picoseconds laser pulses (Fig. 3).

The TIN regime is effective for h_p_ < h_m_ where h_p_ denotes the liquefied thickness of the donor layer within the pulse duration, τ, and h_m_ is the metal layer thickness. h_p_ can be calculated given the optical absorption depth, h_o_, and the heat diffusion depth, h_h_, within the pulse duration. In the case of nano and sub-nano second pulses, the temperature of the lattice and the electrons is the same and a single heat equation governs the layer heating. A good approximation^26^ of the heating depth during the pulse duration is given by 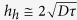 where D denotes thermal diffusion coefficient of the metal. The lower limit for the TIN condition can be estimated by Equation 1 where λ is the laser wavelength and κ is the imaginary part of the index of refraction.


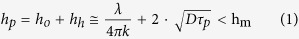


Note that Eq. 1 does not reflect the fact that there is a certain threshold in pulse fluence for jetting to take place. However, TIN-LIFT also depends on pulse fluence reflecting four main printing regimes: 1) Pulse fluence is below threshold and no jetting takes place; 2) Near threshold regime; 3) Stable droplet jetting regime; 4) Sputter regime. At a too low pulse fluence there is not enough energy to allow for material transfer (Regime1), on other hand, when the fluence is too high, extra-energy is invested leading to local explosion of the molten metal layer which results is transfer of the mat erial in the form of a sputter jet (a burst of very small droplets with low directionality). Near threshold (regime 2) the printing process is typically characterized by an unstable jetting. Optimum jetting conditions are obtained above threshold (chap1 supplementary) which, depending on the specifics of the material involved can give large enough working window for quality, single droplet jetting.

The laser pulse fluence, F_p_ has to be high enough to locally liquefy the entire metal layer thickness h_m_ by heat propagation. On the other hand, there is an upper limit to F_p_ in order to avoid unstable deformation and breakup of the thin metal layer. There is therefore a maximal value for the metal layer thickness, h_max_, for which stable transfer of micro-droplets can be supported. For a precise determination of these conditions we have to consider the mechanical and fluid dynamics under high pressure, and to describe the thermal and phase change front propagation. In order to simplify matters and provide a reasonable estimation, without resorting to such 2D numeric simulations, we make the assumption that the temperature in the layer is bounded by the metal boiling temperature (see Supplement). This is a reasonable approximation since above the boiling temperature the pressure generated at the interface would be too large and would result in drastic layer deformation and breakage instead of droplet jetting.

Figure 2 describes the conditions for TIN formation as a function of the layer thickness and the laser pulse duration. It is remarkable that the practical working window for thin-films (thickness ~<1 μm) is obtained for sub-nanosecond pulses. In contrast for short pulses, i.e. of several picoseconds duration and shorter, the working window becomes very narrow. We note that the relevant sub-nanosecond regime with its convenient working window can be served by several laser technologies, e.g. monolithic passive Q-switched lasers and, more recently, high power fiber lasers in MOPA or MOFA configurations.

In order to confirm the TIN regime criterion as depicted in Fig. 2, we carried out LIFT transfer studies four metal layer thicknesses: 300; 500; 750; and 1000 nm. We use for this study is a frequency doubled, Nd:YAG laser (λ = 532 nm), passively Q-switched, with laser pulse durations of 400 ps (Powerchip from *Teem Photonics)*. At this wavelength there is a reasonable absorption by most metals of interest as compared with the fundamental wavelength (1064 nm). Specifically, at 532 nm copper has an absorption of ~40% (depending of the glass substrate). The red diamonds in Fig. 2 represent these four test conditions.

The printed pattern chosen for this study consists of two concentric rings, each with a line width of 35 μm. The radii are 35 μm and 105 μm for the inner and outer rings respectively, as show in the SEM images in Fig. 3(e,f). The rings are made up from overlapped printed metal droplets. The droplet are being jetted from a distance of D_HAZ_ apart, which indicated the thermal heat zone, in order to maintain uniform print quality. A special recipe was used in order to guarantee high packing ratio of the droplets (suppl.). We have printed such patterns using three different gaps: 100, 400 and 1000 μm for each one of the 4 different layer donor thicknesses (thus, twelve printed structures in total). For each donors thickness {300, 500, 750 and 1000} nm we have used a different pulse energy {0.67, 0.88, 0.96, 1.12}J/cm^2^ respectively. The fluence which was chosen for each donor thickness was the one which gave a minimal height variation when jetting from the different gap distances (see Fig. 3a4,b4,c4,d4). A similar method methods used in ref. 27. The structure quality was determined from 3D topography measurements (Contour GT-InMotion interferometeric microscope, Bruker). Figure 3 depicts the 2D contours for the twelve pattern combinations described above, where indices a, b, c and d refer to the donor thicknesses and indices 1, 2 and 3 refer to the gap distances. As expected from the analysis presented in Fig. 2, the printing quality and line definition obtained with the 300 nm donor showed a high dependency on the gap distance; in this case 100 μm is already too large a gap for quality printing.

Maintaining the TIN condition guarantees a clear improvement of printing quality. For the three donors with thickness >300 nm the LIFT printing results are of good quality when the gap is at least 100 μm. We note that for the 500 nm donor the printing is still stable at a gap larger than 400 μm. For thicker donors (750 nm and 1000 nm), we observe a broadening of the printed pattern as a function of the gap, which reflects droplet deformation during flight and spreading as the droplet impacts the substrate. According to the model presented by Fig. 2, we suggest that the spreading for thicker donors (750–1000 nm) is not due to lower positional accuracy (as in the case of 300 nm thick donor; see Fig. 3), but rather due to the larger volume of the droplets, which also gives rise to larger height of the printed patterns.

Figure 4 depicts SEM images of a 300 nm copper donor layer just after the LIFT transfer process. Figure 4a depicts three different pulse fluence levels, F_p_, which correspond to different regimes; (a1) at threshold (F_p_ = 0.6 J/cm^2^); (a2) droplet transfer regime ((F_p_ = 0.67 J/cm^2^); (a3) droplet scattering regime (F_p_ = 0.75/cm^2^). The SEM images reflect well the droplet ejection mechanism, from a liquid pool formation just prior to jetting (a1), to droplet jetting (a2), and the excess pulse energy case giving rise to sputtering (a3). Also note the schematic inset in Fig. 4a1,a2 depicting this liquid pool case. The same study was done using a thicker donor (500 nm copper layer) maintaining the same pulse fluence regimes (b1) F_p_ = 0.74 J/cm^2^; (b2) F_p_ = 0.88 J/cm^2^; (b3) F_p_ = 0.96 J/cm^2^ (Fig. 4b). Here we observe the quasi–nozzle formation during droplet ejection according to the TIN theory as shown above in Fig. 1b. Further support for the TIN-LIFT printing mechanism and to the model schematically described in Fig. 1, are given in references^27^,^28^,^29^ where the droplet spreading^28^, the dynamics of a single droplet crossing the gap^30^, and the high directionality^27^ are discussed and analyzed, particularly the dependence of these quantities on the donor thickness, the donor-to-acceptor gap and laser fluence.

Next, we show how under the TIN-LIFT printing conditions it is possible to generate quite unusual, unsupported, 3D metal structures. The first example, shown in Fig. 5, demonstrates printed metal pillars with an extremely high aspect ratio. This is a clear manifestation of the high positional accuracy of the metal droplets under TIN conditions which allows the droplets to pile up, one on top of the other, with high accuracy. The fast solidification (within nanoseconds) of each molten metal droplet as it lands on the previously printed droplet, prohibits excess spreading and holds the pillar width constant as the structures are being built up. Figure 5a,b are SEM images of copper pillars, each composed of 400 droplets printed from a gap of 300 μm (the gap is maintained as the pillar evolves in height). Figure 5a depicts four pillars where each was printed at a different pulse fluency, F_p_. From left to right: F_p_ = 0.8; 0.84; 0.88; and 0.92 J/cm^2^. For this specific series of pillars, the diameter increases from 7 μm to 10 μm and the height goes from 150 μm to 220 μm. The measured changes in widths and heights reflect the increase in droplet volume as the pulse fluency increases. By approximating the pillars by perfect cylinders we can extract the volume of a single droplet. We found the droplet volume to be in the 10’s femto-litters range: 15fL; 22fL; 34fL; and 45fL respectively, for the four fluencies indicated above.

It is possible to take advantage of the fast solidification rate of femto-litter droplets to print various bent pillars and unsupported curved structures. Still, the curvature is necessarily limited by the solidification rate and will depend on the droplet volume. For example, for 45fL droplets we find that the bent angle can go up to 30°. This is demonstrated in Fig. 5(c) for pillars printed in two steps. First, an upright pillar part composed of 100 droplets was printed. Then, 100 droplets where deposited, with each additional droplet shifted by a distance ΔD from its predecessor (ΔD = 0, 0.1, 0.2, 0.3, 0.4 μm, from left to right, respectively). The repeatability and the accuracy of this printing regime were then demonstrated by the rightmost pillar in Fig. 5(d). This figure depicts a pillar with two consecutive deflection angles, where 50 droplets were first printed with zero shift, ΔD = 0, followed by 50 droplets with ΔD = 0.3 μm and finally 100 droplets with ΔD = −0.3 μm.

Such metal pillars can find various emergent applications in photonics^31^,^32^, biology^33^, micro-mechanics^34^,^35^ and micro-batteries^9^,^36^. For example, there is a growing need for tailored made electrodes for brain activity measurements or specific neural activation^37^,^38^. The printing method described here allows fabricating tailored micron-scale arrays where each electrode has a pre-determined position, a special length and orientation. This amounts to fabricating 3D electrode arrays with design specificity which is hard to obtain by current fabrication methods. Further opportunities might be opened by the specific choice of the electrode metal or building composite metal structures. In a similar manner one can envisage the potential use of this printing method to fabricate porous, micron-scale, dense electrodes for micro-batteries printed on various, also sensitive, substrates. We refer the reader to the Supplement to this article for more details on applications and print material properties.

We note that in general, metal pillars are currently manufactured using conventional integrated circuit (IC) processes which are limited to specific materials compatible with IC processes. Also, the height of the electroplated or evaporated columns is limited to a few tens of microns only. There are a few digital printing methods, i.e. inkjet, super-inkjet^9^, molten metal jet^39^,^40^ which can be used for metal buildup and with LIFT^30^. However, these methods are limited to a particular range of materials and have a much lower horizontal resolution than that obtained by IC processes. Remarkable point is that such a LIFT method has no particular requirement on ambient conditions for printing.

In the next example, we show how this method can provide arbitrary 3D structures. The fabrication process conforms to standard 3D printing and consists of overlapping micron-scale copper droplets. Figure 6 depicts three concentric cylinders with exceptionally high aspect ratios, a height of 220 μm and wall thickness of 25 μm, 35 μm and 45 μm for the inner, middle and outmost cylinders, respectively.

When jetting molten metal micro-droplets under ambient atmospheric conditions there is a risk of the droplet gets oxidized. Such oxide layers which form on the droplet surface will affect the electrical and mechanical properties of the printed structures. We have found however that under the printing conditions we were using, there is no oxidation observed for either copper or gold all across the working window. In contrast, oxidation is evident when printing aluminum; (see supplementary section for more details). The degree of oxidation in this case depends strongly on the donor-to-acceptor gap and laser power density. Other physical parameters of LIFT printed metals have been determined, such as the electrical resistivity of copper, *ρ* = 3.6 × bulk copper (mono-crystalline Cu), which is due mainly to the multi-crystallinity of printed copper^41^, composed of nano-grains with grains sizes down to 20 nm and to some extent also to the presence of nanometric voids. In the supplement we provide more information on additional physical and morphological properties of TIN-LIFT printed metal structures.

The third example demonstrates how mixed-material 3D structures can be printed by the TIN-LIFT method. In fact, a large variety of solids can be printed using this method provided the printing parameters are properly chosen according to Equation 1. In Fig. 7 we demonstrate a bi-metal object composed of a slab of copper (370 × 370 × 15 μm) and a gold comb printed on top. The comb’s fingers width is 25 μm and the height is 15 μm. The gold droplets were printed from a gold donor layer 700 nm thick. We note that to manufacture such a structure using conventional IC methods would involve a large number of process steps in a hazardous environment. LIFT printing of various metals will typically require a rather similar set of parameters. In our laboratory, we have already printed aluminum, gold, silver and various copper alloys, and we have found all to behave according to the same TIN-LIFT criterion.

It can be observed from Fig. 7(c,e) that the printed gold tracks show a more porous structure than the copper base slab. It is due to the fact that we have used the same conditions, similar laser pulse parameters and layer thickness, for both metal, although the density of gold is much higher and would require tuning the printing conditions for optimal results. These results exemplify another advantage of the TIN method, namely that one can control the printed material porosity by varying the overlap between droplets as well as their volume and jetting speed by using appropriate printing recipes. For example, it could even be possible to print a 3D object with an anisotropic density. The capacity to engineer materials properties in all three dimensions at the voxel (single droplet) scale is of the highest research priority in the field of materials engineering^42^,^43^.

In summary, we have shown that there is a LIFT printing regime which allows stable jetting of femto-litter metal droplets from a large gap between the donor layer and the receiver substrate. This allows for a wider use of LIFT printing technology, also in 3D material printing and high-resolution functional digital printing in general. Such stable jetting arises from a dynamically formed quasi-nozzle in the metal layer as a result of a well-tuned heat transfer process. We have termed this the “thermal induced nozzle” (TIN) method in LIFT printing. We have demonstrated the TIN regime using sub-nanosecond duration laser pulses and metal donor layers <1 μm thick in accordance with the theoretical prediction we have presented. There is a clear advantage to build up metal structures by jetting molten metal micro-droplets, since, unlike e.g. dispensing or ink-jetting, the ultra-fast solidification rates guarantee that wetting related issues upon landing are negligible. This allows for printing high-resolution, ultra-high aspect ratio structures as well locally tuning the printed material density. Another important advantage is a rather large variety of printable materials, metals and dielectrics, compatible with this printing method. It is mainly due to the simplicity of donor preparation and the generality of the jetting process. This is in striking contrast to the limited choice of materials when it comes to printing metal inks/pastes. The TIN-LIFT regime allows for the fabrication of multi-material, composite structures at the voxel/droplet level. The authors believe that this particular ability of the method will open the route to the fabrication of new advanced material and meta-materials.

## Additional Information

**How to cite this article**: Zenou, M. *et al.* Laser jetting of femto-liter metal droplets for high resolution 3D printed structures. *Sci. Rep.* **5**, 17265; doi: 10.1038/srep17265 (2015).

## Supplementary Material

Supplementary Information

## Figures and Tables

**Figure 1:**
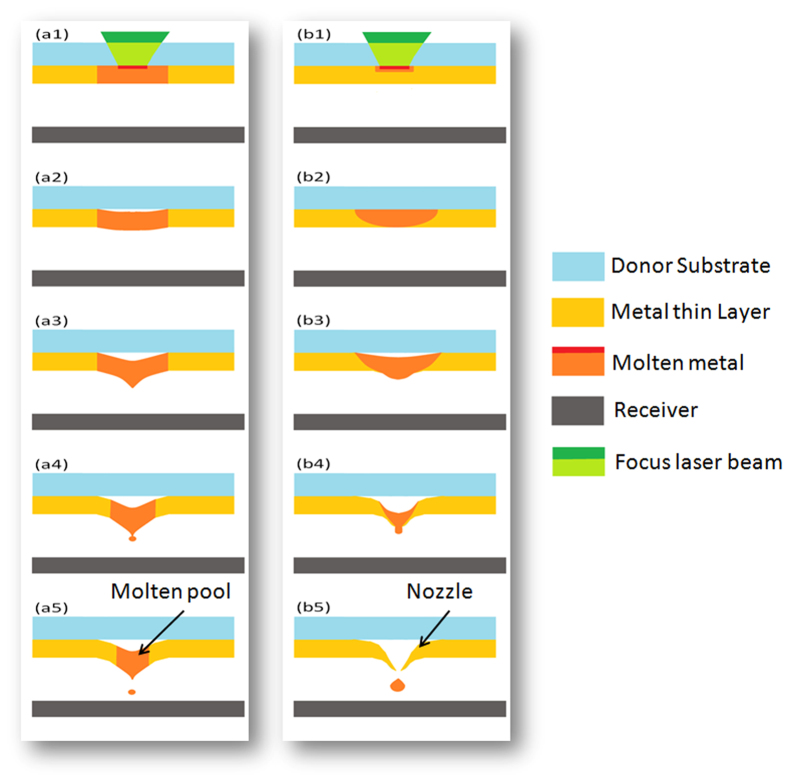
(**a1**–**a5**) A schematic illustration of the evolution that takes place in the classic case of “melt pool” LIFT transfer. The metal layer melts completely within the pulse duration. (**b1**–**b5**) Schematic of the TIN transfer evolution where only part of the metal layer gets melted within the pulse duration.

**Figure 2:**
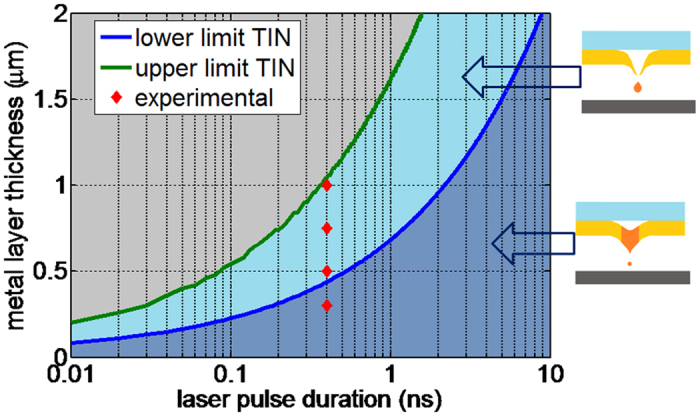
TIN criterion for copper metal plotted as a function of the laser pulse duration and the copper layer thickness. The lower limit curve (blue) is obtained from Eq. 1 with D = 1.1 cm^2^/s, λ = 532 nm and κ = 2.59. The upper limit (green curve) is obtained using Eq. 2 (see Supplement). The experimental results (red diamonds) obtained under conditions described in relation to in Fig. 3.

**Figure 3:**
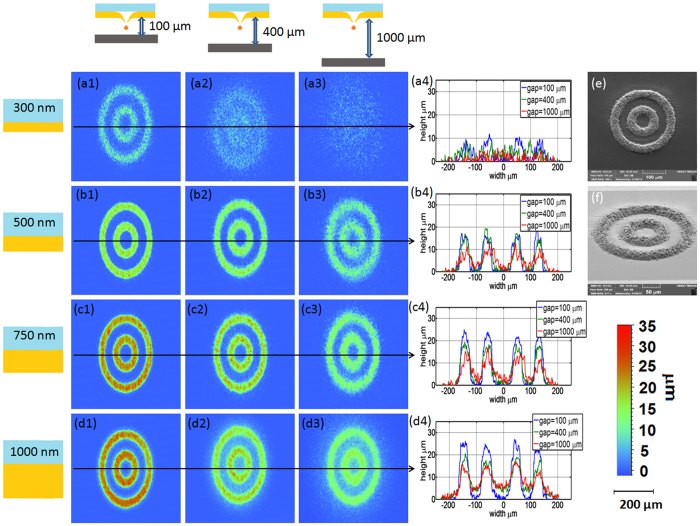
3D contours of the metal concentric rings printed on soda lime coated with a thin layer of 100 nm gold. Donor layer thicknesses: (**a**) 300 nm, (**b**) 500 nm, (**c**) 750 nm and (**d**) 1000 nm, and gap distances of (1) 100 μm, (2) 400 μm, and (3) 1000 μm. (**a4**–**d4**) Superimposed line profiles for different gap distance and for each one of the donor thickness (the profiles were taken across the middle of the rings, see black arrows plotted over the 3D contour images which indicate the line profile direction); (**e**) SEM image of the concentric rings as printed under conditions (**b**2); (**f**) Same SEM image at a tilt of 55 degrees.

**Figure 4:**
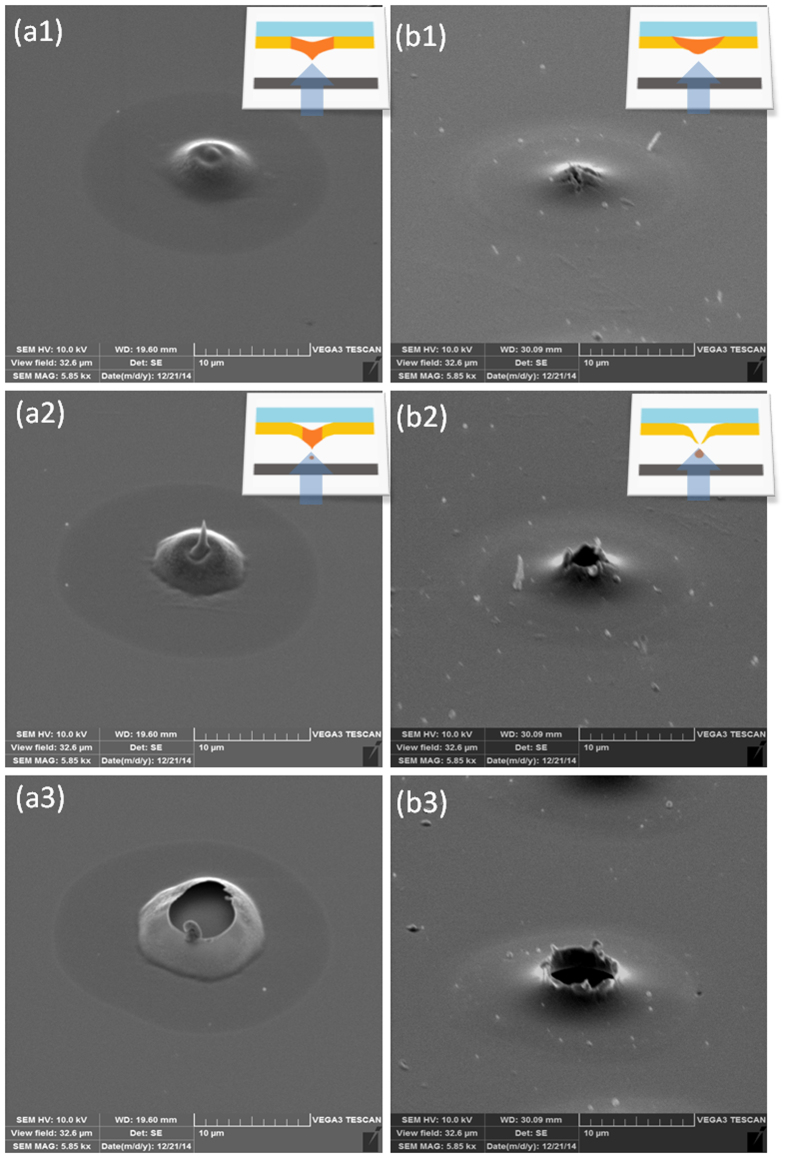
SEM images of the donor layers after LIFT sessions under different conditions. (**a**) Donor copper layer is 300 nm thick: (**a1**) pulse fluence, F_p_ = 0.6 J/cm^2^ (just below the jetting threshold); (**a2**) optimal droplet jetting fluence, F_p_ = 0.67 J/cm^2^; (a3) F_p_ = 0.75 J/cm^2^ (excess energy resulting in scattered jetting regime). (**b**) A copper donor layer 500 nm thick: (**b1**) F_p_ = 0.74 J/cm^2^ (below printing threshold); (**b2**) optimum droplet transfer energy, F_p_ = 0.88 J/cm^2^;(**b3**) F_p_ = 0.94 J/cm^2^ (scattered printing regime).

**Figure 5:**
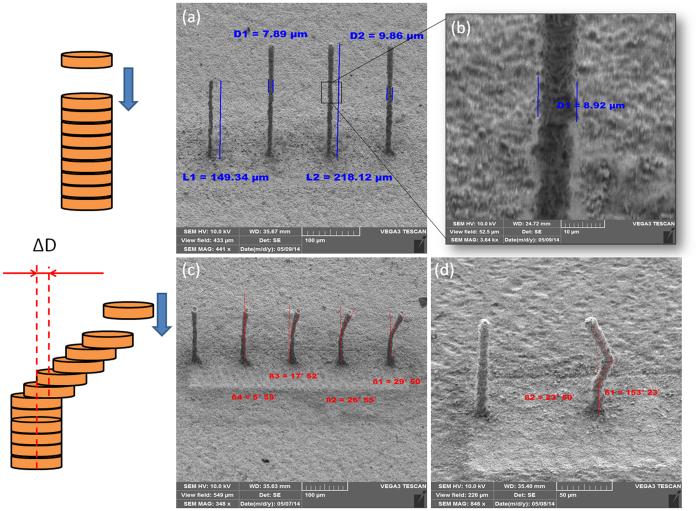
(**a**) SEM image of printed pillars each made up by a pileup of 400 droplets printed on a copper foil. The four pillars were printed at a different pulse fluence, from left to right: F_p_ = 0.8; 0.84; 0.88; and 0.92 J/cm^2^. (**b**) A zoom in on the third pillar from left of (**a**,**c**) SEM image of two-segment, bent pillars; 100 droplets each, with shift ΔD = 0, 0.1, 0.2, 0.3, 0.4 μm (left to right). (**d**) SEM image of an upright pillar and a three-segment bent pillar for which the 2^nd^ segment is composed of 50 droplets, each shifted by ΔD = 0.3 μm, and in the 3^rd^ segment there are 100 droplets each shifted by ΔD = −0.3 μm.

**Figure 6:**
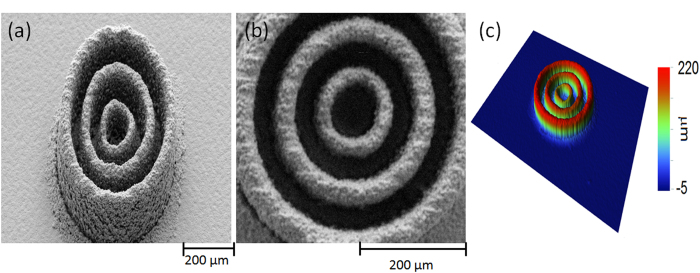
(**a**) SEM image of three concentric cylinders printed on a copper foil, with a height of 220 μm and wall thickness: 25 μm, 35 μm and 45 μm for the inner, middle and outer cylinders, respectively (SEM image taken at 35° tilt); Minor density of debris and scattered material around the pattern can also be observed. (**b**) Top-view, zoom-in image of (**a)**; (**c**) a 3D measurement of the same sample.

**Figure 7:**
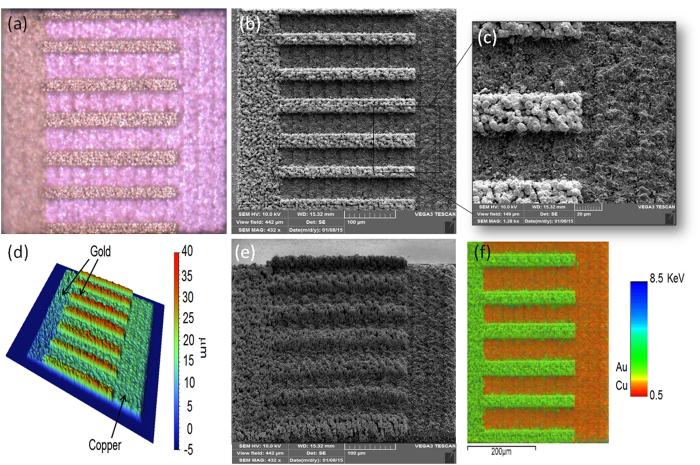
A TIN-LIFT printed multi-material object printed on soda lime coated with a thin layer of 100 nm gold. At the bottom a copper slab (370 × 370 × 15 μm) and on top a gold comb (gold fingers 25 μm wide and 15 μm thick). (**a**) An optical microscope image, (**b**) an SEM image (top view), (**c**) an SEM image (zoom-in on fingers part), (**e**) a 3D optical microscope topography image, (**f**) an SEM image at 45° tilt, (**g**) an EDS atomic composition map.

## References

[b1] Seung H. Ko. *et al.* All-inkjet-printed flexible electronics fabrication on a polymer substrate by low-temperature high-resolution selective laser sintering of metal nanoparticles. Nanotechnology 18, 345202 (2007).

[b2] Arias A. C. *et al.* All jet-printed polymer thin-film transistor active-matrix backplanes. Appl. Phys. Lett. 85, 3304 (2004).

[b3] Sirringhaus H. *et al.* High-Resolution Inkjet Printing of All-Polymer Transistor Circuits. Science 290, 2123–6 (2000). 11118142 10.1126/science.290.5499.2123

[b4] Zschieschang U., Klauk H., Halik M., Schmid G. & Dehm C. Flexible Organic Circuits with Printed Gate Electrodes. Adv. Mater. 15, 1147 (2003).

[b5] Gamerith S. *et al.* Direct ink-jet printing of Ag–Cu nanoparticle and Ag precursor based electrodes for OFET applications. Adv. Funct. Mat. 17, 3111–3118 (2007).

[b6] Lee Y., Choi J., Jong Lee K., Stott N. E. & Kim D. Large-scale synthesis of copper nanoparticles by chemically controlled reduction for applications of inkjet-printed electronics. Nanotechnology 19, 19415604 (2008). 10.1088/0957-4484/19/41/415604 21832649

[b7] Rapp L., Ailuno J., Alloncle A. P. & Delaporte P. Pulsed-laser printing of silver nanoparticles ink: control of morphological properties. Opt Express 19(22), 21563–74 (2011). 22109005 10.1364/OE.19.021563

[b8] Zenou M., Winter S., Sa’ar A. & Kotler Z. Laser-Forward-Transfer of Metal NP Ink Droplets: Parametric Analysis. Nanosci.&Nanotech. Letters 5, 1–4 (2013).

[b9] Ho C., Murata K., Steingart D. A., Evans J. W. & Wright P. K. A super ink jet printed zinc–silver 3D microbattery. J. Micromech. Microeng. 19,094013 (2009).

[b10] Kashiwagi Y. *et al.* Direct transparent electrode patterning on layered GaN substrate by screen printing of indium tin oxide nanoparticle ink for Eu-doped GaN red light-emitting diode. Appl. Phys. Lett. 105, 223509 (2014).

[b11] Nuzhnyy D. *et al.* Percolation in the dielectric function of Pb(Zr, Ti)O3 – Pb2Ru2O6.5 ferroelectric –metal composites. J. Phys. D: Appl. Phys. 47,495301 (2014).

[b12] Shuangyu L. *et al.* Nitrogen-doped reduced graphene oxide for highperformance flexible all-solid-state microsupercapacitors. J. Mater. Chem. A 2, 18125–18131 (2014).

[b13] Krebs F. C., Espinosa N., Hösel M., Søndergaard R. R. & Jørgensen M. 25th Anniversary Article: Rise to Power – OPV-Based Solar Parks. Adv.Mat. 26, 1, 29–39 (2014). 10.1002/adma.201302031 24741693

[b14] Hübler A. *et al.* Printed Paper Photovoltaic Cells. Adv. Ener. Mat. 1, 6, 1018–1022 (2011).

[b15] Arved C. H. *et al.* Fully mass printed loudspeakers on paper. Organic Electronics 13, 2290–2295 (2012).

[b16] Zergioti I. *et al.* Microdeposition of metal and oxide structures using ultrashort laser pulses. Appl. Phys. A 66, 579–582 (1998).

[b17] Willis D. A. & Grosu V. Microdroplet deposition by laser-induced forward transfer. Appl. Phys. Lett. 86, 244103 (2005).

[b18] Willis D. A. & Grosu V. The effect of melting-induced volumetric expansion on initiation of laser-induced forward transfer. Appl. Surf. Sci. 253, 4759–4763 (2007).

[b19] Bohandy J., Kim B. F. & Adrian F. J. Metal deposition from a supported metal film using an excimer laser. J. Appl. Phys. 60, 1538 (1986).

[b20] Mogyorósi P., Szörényi T., Bali K., Tóth Z. & Hevesi I. Pulsed laser ablative deposition of thin metal films. Appl. Surf. Sci. 36, 157–163 (1989).

[b21] Narazaki A., Sato T., Kurosaki R., Kawaguchi Y. & Niino H. Nano- and Microdot Array Formation of FeSi2 by Nanosecond Excimer Laser-Induced Forward Transfer. Appl. Phys. Exp. 1, 057001 (2014).

[b22] Banks D. P., Grivas C., Mills J. D., Eason R. W. & Zergioti I. Nanodroplets deposited in microarrays by femtosecond Ti:sapphire laser-induced forward transfer. Appl. Phys. Lett. 89, 193107 (2006).

[b23] Zywietz U., Evlyukhin A. B., Reinhardt C. & Chichkov B. N. Laser printing of silicon nanoparticles with resonant optical electric &magnetic responses. Nature Com. 5, 3402 (2014). 10.1038/ncomms4402 24595073

[b24] Kuznetsov A. I., Unger C., Koch J. & Chichkov B. N. Laser-induced jet formation and droplet ejection from thin metal films. Appl Phys A 106, 479–487 (2012).

[b25] Unger C., Koch J., Overmeyer L. & Chichkov B. N. Time-resolved studies of femtosecond-laser induced melt dynamics. Opt. Exp. 20, 22, 24864 (2012). 10.1364/OE.20.024864 23187253

[b26] Chichkov B. N., Momma C., Nolte S., Alvensleben F. Y. & Tiinnermann A. Femtosecond, picosecond &nanosecond laser ablation of solids. Appl. Phys. A 63, 109–115 (1996).

[b27] Zenou M., Sa’ar A. & Kotler Z. Digital laser printing of aluminum microstructure on thermally sensitive substrate. J. Phys. D: Appl. Phys. 48, 205303 (2015).

[b28] Zenou M., Sa’ar A. & Kotler Z. Laser Transfer of Metals and Metal Alloys for Digital Microfabrication of 3D Objects. Small, doi: 10.1002/smll.201500612 (2015) 25966320

[b29] Zenou M., Sa’ar A. & Kotler Z., Supersonic laser-induced jetting of aluminum micro-droplets. Appl. Phys. Lett. 106, 181905 (2015).

[b30] Visser C. W. *et al.* Toward 3D Printing of Pure Metals by Laser-Induced Forward Transfer. Adv. Mater. 27, 4087–4092 (2015). 26045211 10.1002/adma.201501058

[b31] Joannopoulos J. D. *et al.* Photonic crystals: putting a new twist on light. Nature 386, 143 (1997).

[b32] Grigorenko A. N. *et al.* Nanofabricated media with negative permeability at visible frequencies. Nature 438, 335–338 (2005). 16292306 10.1038/nature04242

[b33] LaVan D. A., McGuire T. & Langer R. Small-scale systems for in vivo drug delivery. Nature Biotechnology 21, 1184–1191 (2003). 10.1038/nbt876 14520404

[b34] Hwan Ko S., Chung J., Hotz N., Nam K. H. & Grigoropoulos C. P. Metal nanoparticle direct inkjet printing for low-temperature 3D micro metal structure fabrication. J. Micromech. Microeng. 20, 125010 (2010).

[b35] Ahn B. Y. *et al.* Omnidirectional Printing of Flexible, Stretchable, and Spanning Silver Microelectrodes. Science 323, 1590–92 (2009). 19213878 10.1126/science.1168375

[b36] Sun K. *et al.* 3D Printing of Interdigitated Li-Ion Microbattery Architectures. Adv. Mater. 25, 4539–4543 (2013). 23776158 10.1002/adma.201301036

[b37] Alivisatos A. P. Nanotools for Neuroscience and Brain Activity Mapping. ACS Nano 7(3): 1850–1866 (2013). 23514423 10.1021/nn4012847 PMC3665747

[b38] Robinson J. T. *et al.* Vertical Nanowire Electrode Arrays as a Scalable Platform for Intracellular Interfacing to Neuronal Circuits. Nature Nanotechnology 7, 180–184 (2012). 10.1038/nnano.2011.249 PMC4209482 22231664

[b39] Lass N., Riegger L., Zengerle R. & Koltay P. Enhanced Liquid Metal Micro Droplet Generation by Pneumatic Actuation Based on the StarJet Method. Micromachines 4, 49–66 (2013).

[b40] Bohandy J., Kim B. F. & Adrian F. J. Metal deposition from a supported metal film using an excimer laser. J. Appl. Phys. 60, 1538 (1986).

[b41] Mayadas A. F. & Shatzkes M. Electrical-Resistivity Model for Polycrystalline Films: the Case of Arbitrary Reflection at External Surfaces. Phys Rev. B 1, 1382 (1970).

[b42] Schaedler T. A., Jacobsen A. J. & Carter W. B. Toward Lighter, Stiffer Materials. Science 431, 6151, 1181–1182 (2013). 24031005 10.1126/science.1243996

[b43] Schaedler T. A. *et al.* Ultralight Metallic Microlattices. Science 334, 6058, 962–965 (2011). 22096194 10.1126/science.1211649

