# Supplementary material for: Laser jetting of femto-liter metal droplets for high resolution 3D printed structures
Source: Sci Rep. 2015 Nov 25;5:17265. doi: 10.1038/srep17265 (PMC4658554; doi:10.1038/srep17265)
Supplement: Supplementary Information [file srep17265-s1.doc]

Supplementary Section:

**Laser jetting of femto-liter metal droplets for high resolution 3D printed structures**

M. Zenou,^1,2^ A. Sa’ar,^2^ and Z. Kotler^1^

1. **Working Window of TIN-LIFT: The role of pulse fluence**

We consider below the printability working window for the TIN-LIFT jetting regime with regard to the pulse energy (or rather the laser pulse fluence, F[J/cm^2^]). We first fix the working conditions, namely donor layer thickness, the distance from donor to acceptor (the gap) and laser pulse duration (400ps). When printing a 3D structure, and in particular a high aspect ratio structure, the measured aspect ratio will strongly depend on the laser pulse energy reflecting three principal printing regimes: 1) near threshold; 2) Stable droplet regime; 3) Sputter regime. Figure S1 manifests these three regimes. Below threshold there is not enough energy to drive the jetting and no material is being transferred. At threshold (F_th_~0.76J/cm^2^) the jetting is rather unstable due to even minor variations in pulse energy, donor thickness and surface imperfections (dirt?). The droplets’ volume at threshold is smaller and prone to deviations in jetting direction. As the fluence increases a stable droplet jetting regime sets in characterized by a high transfer directionality which allows for the fabrication of ultra-high-aspect-ratio structures. Figure S1 depicts the profiles of pillars each obtained by deposition of 40 droplets. The profiles obtained by 3D optical microscopy. Recall that figure 5 in the article we present pillars made up of 400 droplets. Note that there is practically no widening of the pillars as droplets are accumulated from 40 to 400 droplets. Such piling up can be carried further on, and in fact we managed to print pillars as high as ~700 µm maintaining the same ~10 µm width. This can be understood by the fact that the each additional droplet matches with quite high precision the previous one with minimal width broadening (see below). This capability is unique to the TIN-LIFT printing method and as far as we know such high aspect ratio, micron-scale, metal geometries has no precedence. It is even more remarkable that such fabrication method is carried out in ambient environment which simplifies matters considerably, also one can print various metals using the same technology ( gold and aluminum[40] for example and a alloys[39]).


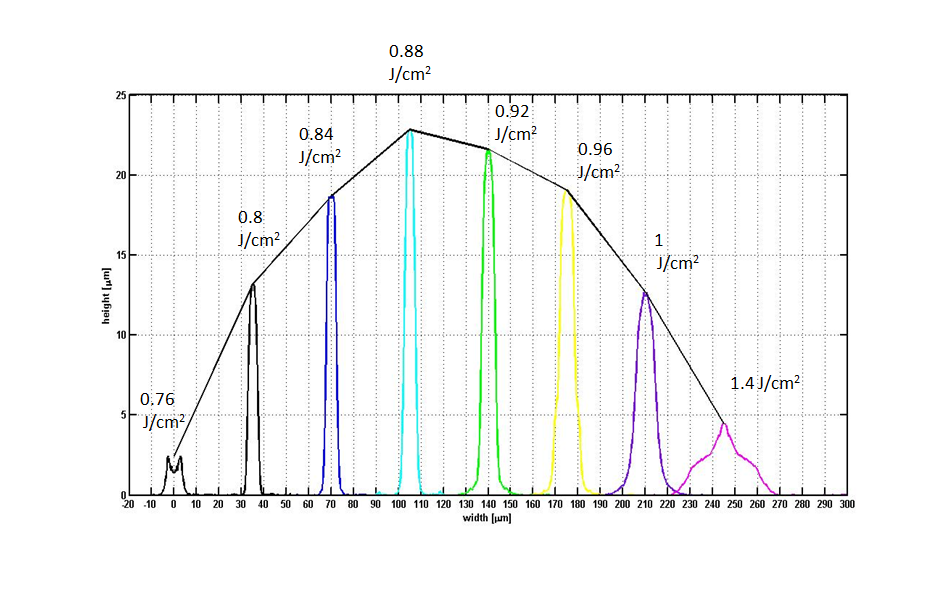
Further increasing the fluence, from 0.8 to 0.9 J/cm^2^, which amounts to jetting droplets of a larger volume, we observe a steady increase in the pillar height and a minor width broadening. We note that the height increase is much more pronounced, due to the instantaneous cooling and solidification of the landing droplets (see next section). This is also manifested in Fig S2 where we depict the pillars height and width (the width is taken at half maximum height) vs. the laser fluence. As the fluence increases beyond ~0.9 J/cm^2^ the jetting loses stability and the main droplet is accompanied by satellites. This is reflected in the broadening deposit which surrounds the pillar base. At a still higher fluence, F~1.4 J/cm^2^, the dominant jetting mechanism has changed predominantly to sputtering, characterized by a spraying jet of very small droplets with low directionality. Here the high local pressure and temperature result in an explosion of the molten material and breakdown into a multitude of smaller droplets. The print geometry will now strongly depend on the gap size, and as the spray has a low directionality there is an increased broadening when the gap gets larger. While the print resolution degrades in the sputter regime, the pattern surface quality improves as it gets smoother. Also, the volume of the transferred material per pulse is higher.

***Figure S1****: Profiles of printed pillars each made up of 40 droplets. Each pillar is printed at a different fluence as indicated.*


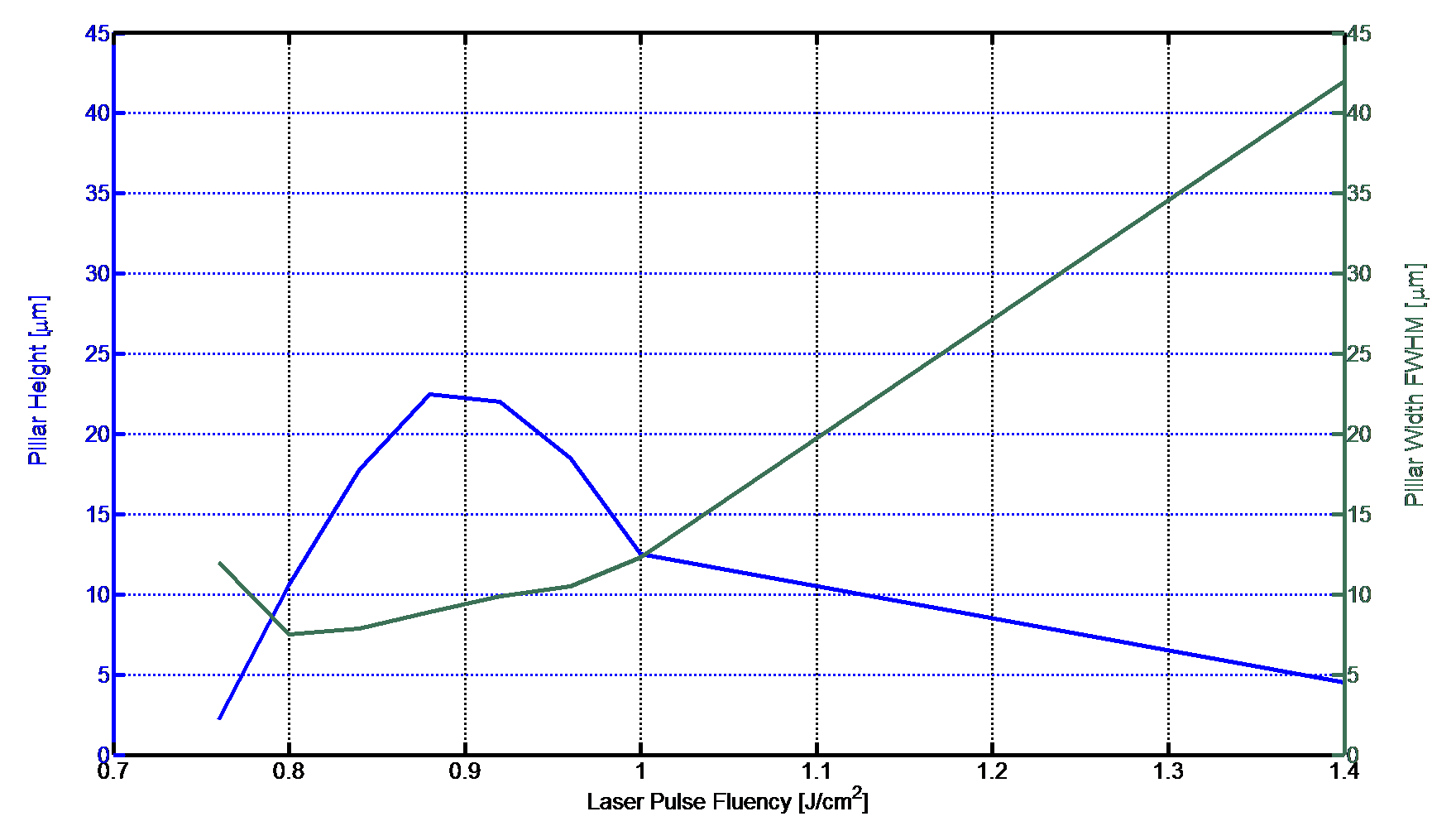


***Figure S2****: Pillars height and width as a function of laser fluence (jetting parameters are the same as in S1)*

Note that the profile shown in figure S1 is a radial average obtained from the 3D data. Figure S3 illustrates the transition from the 3D measurement of the pillar to the radially averaged result.

***
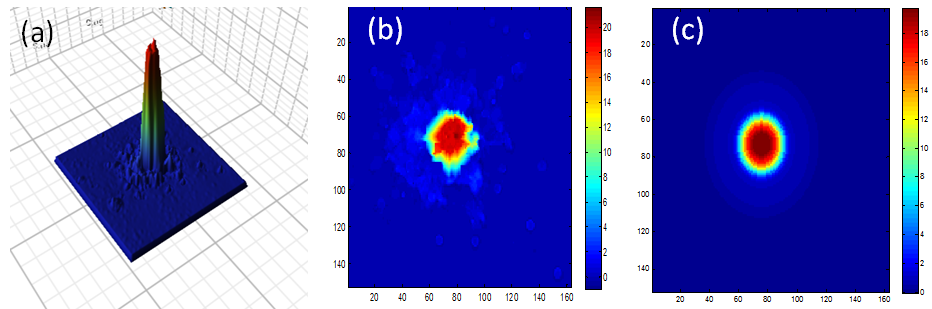
***

**Figure S3:** (a) 3D measurement of a pillar printed at a fluence F=0.92 J/cm^2^; (b) 2D contour plot of the same data loaded into a Matlab program and processed to give the mean radial contour from (c).

1. **Metal droplet solidification rate**

In the previous section we have seen that an increase in droplet volume which results from higher fluence affects more the pillars height growth rate than its width. Moreover, the capability to print bent pillar structures (see (Fig. 5c) indicates clearly that the landing molten metal droplets cool down and solidify instantly. To modeled the thermal evolution of the droplet using a finite element simulation (*COMSOL* - thermal simulations package). Specifically we modeled the solidification rate of a circular copper disk on a solid copper substrate initially at room temperature. For a model copper droplet of 20fL (a copper disk of xx diameter and yy height) the solidification time is less than 10 ns. The simulation results are shown in figure S4. The solidification time is evaluated at less than 10 ns when the while cooling to room temperature takes ~1 µs. We can therefore conclude that for a printing rate <1MHz no effect should be expected on the pillar morphology (Fig. 5) due to print rate. On the other hand, we expect the effective printing rate to be limited by the overall jetting duration which is ~<2us (=incubation time+ jetting + landing time ~< 2us [28].


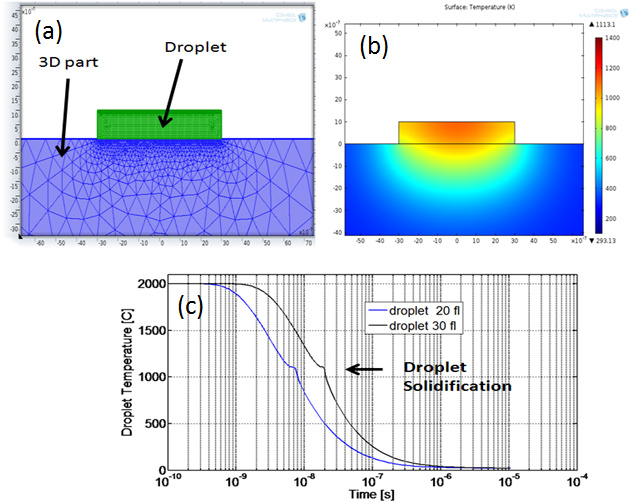


***Figure S4:*** *(a) Scheme of the nonlinear mesh for substrate and droplet used in the COMSOL simulation; (b) Temperature distribution at t=1ns; (c) Droplet temperature in function of the time.*

1. **Line Printing**

The printing of 2D horizontal patterns consists of overlapping droplets printed next to each other. Using the TIN-LIFT method we can print high resolution conductors with tailored sheet resistance by tuning the height of the pattern. Figure S5 depicts a 7 µm wide and 10 µm thick printed gold. This line was printed by setting the distance between overlapping printed droplets at 3 µm.


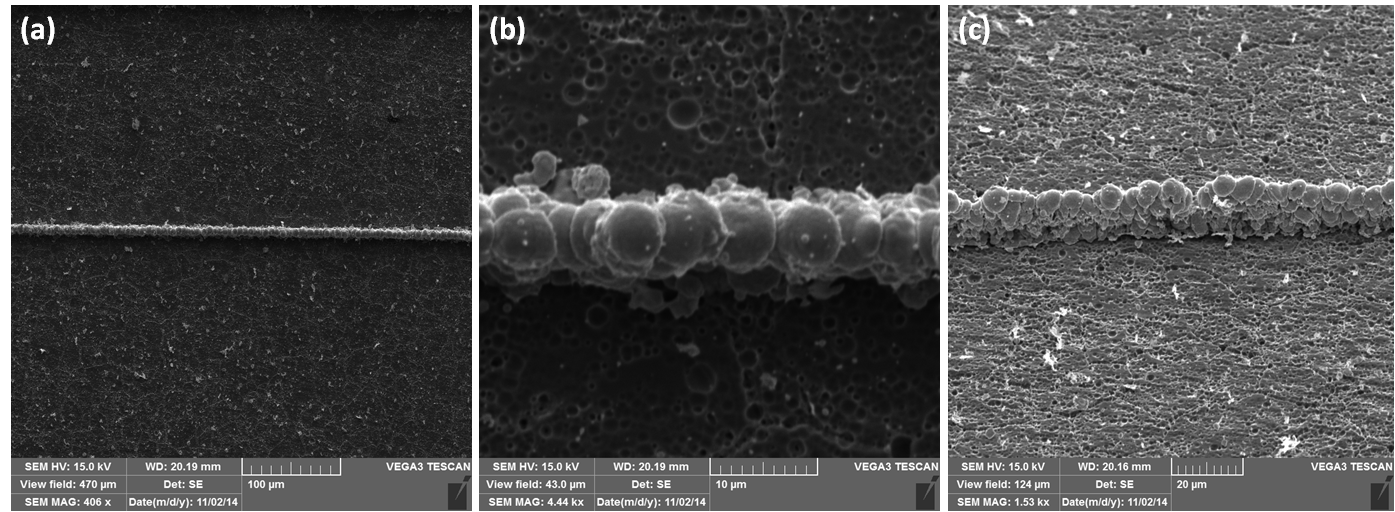


***Figure S5****: (a) SEM image of LIFT printed gold line; (b) a magnified view; (c) A tilted view (35^o^).*

1. **Porosity of Printed Metal Structures**

We evaluated the porosity of TIN-LIFTed metal structures by printing copper tracks on epoxy laminate on top of which there is a typical electroplated copper pattern. The LIFT printed copper track partially coats the electroplated line (see Fig S6). This will serve as a reference when considering the metal morphology. A FIB cross section was carried out at the junction between the electroplated and LIFTed copper track (Fig S6). Random nanometric voids can be seen which we evaluated as less than 4% of the printed volume as determined by image processing. It is evident from the print morphology that each droplet lands in a molten state and conforms to the shape of the metal substrate before fully solidifying. The rapid droplet solidification is in some cases too short, and wetting is not complete leading to appearance of nanometric voids in the structure. Note also that the LIFT printed track accurately follows along the electroplated copper step in a rather perfect match. The air gap between the two structures is due to copper oxide which prevents adhesion. Finally we should note that by properly choosing the printing parameters, for example the gap size and the droplet overlap, it is possible to control the structure porosity and also increase it for certain applications. This could possibly be beneficial for applications where partial transmission of liquids or gas through a metal wall is required, e.g. for drug delivery, or otherwise where enhanced surface is needed, as for example in battery electrodes.


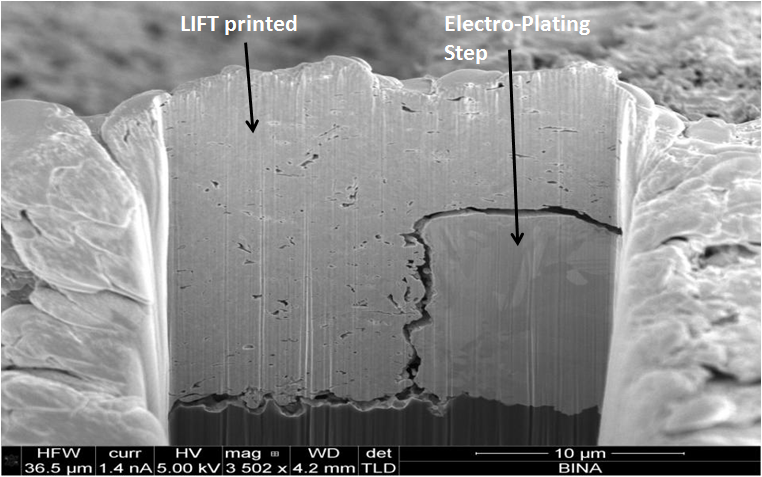


***Figure S6****: A FIB cross section of LIFTed copper line in a conformal manner along the step of an electroplated copper track. The nanometric voids in the LIFT printed copper are evident.*

There is no indication that re-melting is taking place in the interface between the droplets (at least to the level of the SEM resolution which is <3 nm). In order to further evaluate such possible effect we have made a FIB cross section in the printed copper structure and imaged the sample with a HR-SEM. A typical result is shown in figure S7, where the absence of grains continuity across the droplets boundary indicates that indeed no re-melting took place. It is evident that each droplet has its unique grain structure also near the boundary.


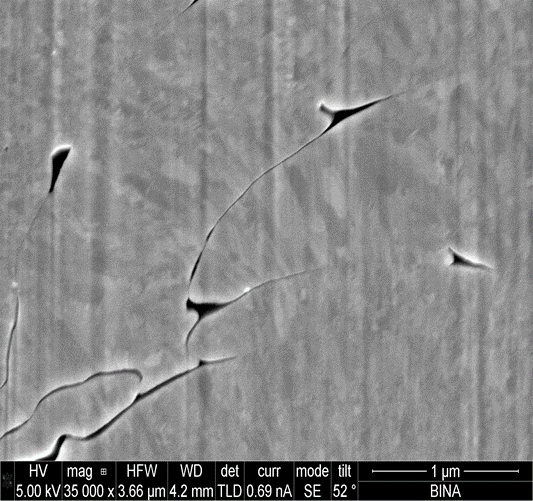


**Figure S7:** HR-SEM of a FIB cross section of printed copper line where the droplet boundaries and grain structure is evident.

1. **Oxidation effects**

An EDS analysis of copper droplets printed under normal atmospheric conditions has shown no indication for oxide (See Fig. S8). We used for this analysis an advanced EDS (Xmax, Oxford Instruments) installed in a Magellan 400L HR_SEM (FEI) which can be used at a low e-beam acceleration voltages and has a tolerance better than 0.5%. The measurement was done at 5 kV with a current of 2nA. While the absence of oxide is quite remarkable given the known reactivity of copper, we attribute it to the fact that the jetting time is very short, <1 μsec (this includes droplet incubation time, droplet travel time, solidification and cooling [40]. The FIB cross-section image shown in figure S3 supports this conclusion as no trace of copper oxide was observed. Moreover, as copper typically shows poor adhesion to copper-oxide, our measurements which have shown excellent adhesion of such printed structures are in support of the above conclusion that no oxidation takes place during the printing process.

It is also well known that the reactivity of copper nano-inks is holding back the wider use of digital printing based on such inks. LIFT printing of copper is an attractive alternative which also simplifies the process steps involved in preparing nana-inks, then printing and sintering. Instead, a single process step is proposed for digital printing of copper in ambient conditions on thermally sensitive substrates such as needed for plastic electronics and 3D hybrid printing.


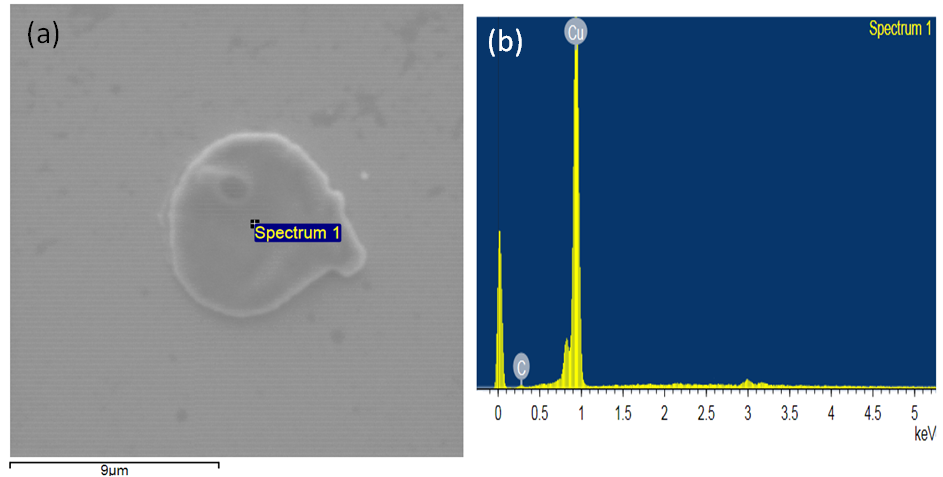


***Figure S8****: (a) A single LIFT printed copper droplet which served for the EDS analysis; (b) EDS spectrum.*

1. **EDS of multi-material prints**

We show below the EDS result which was measured on the copper/gold sample which we present in figure 7 of the article. Figure S9 shows that in the indicated region of interest (ROI) for which the EDS was taken for either copper or gold, the EDS analysis shows the presence of only the respective metal. We should note here that the capacity to print multi-material structures where at the voxel level a specific type of metal can defined and printed will wide open many opportunities in design and fabrication of new complex materials from graded structures to entirely new multi-metal compositional structures and active devices.

***Figure S9****: (a) Two ROI areas for EDS, one for copper and one for gold indicated on the SEM image of the gold on copper structure; (b)EDS spectra: red scan for copper and yellow for gold.*


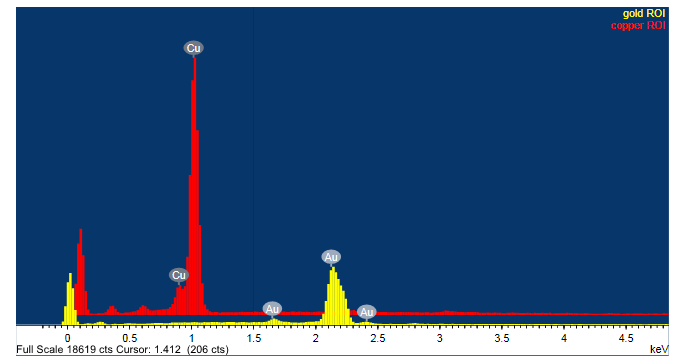


(a)

(b)

1. **Calculation of the upper limit curve shown in Figure 2:**

We calculate below the upper limit curve which was shown in Figure 2 of the article. Together with Eq. 1 it defines the TIN-LIFT working window for varying pulse widths and donor layer thickness. We make the assumption that the maximum energy, E_ma_, is that energy which will get the metal layer to its boiling point within the pulse duration (Eq. 1 in the article). The threshold energy E_th_(h_m_) for jetting of a metal droplet is the energy required to melt the metal layer through its entire thickness, h_m_. The maximum metal layer thickness, h_max_, which can then be melted is obtain from E_max_=E_th_(h_max_). E_max_ is then given by Eq. 2:

(2) here D_L_ is the laser spot diameter (at 4σ), T_melt_ = 1357 K and T_boiling_ = 2835 K are the copper melting and boiling temperatures, respectively, H_m_=207 J/g is the latent heat of fusion, H_b_=205 J/g is the latent heat of evaporation, c_p_~ c_pl_ = 0.386 J/gK is the solid and liquid heat capacities, and ρ~ρ_l_ =11.1 g/cm^3^ is the solid and liquid phase density. The threshold energy E_th_ is given by:

(3)

Where Dm is the lateral diameter of the liquefied metal spot assuming the lateral heat propagation is approximately equal to the layer thickness.

(4)

The upper limit is deduced from the pulse fluence required for the donor material to reach boiling temperature within the pulse duration. This condition comes from the fact that heated layer when reaching the boiling condition will explode. From the calculation of the energy Emax one can deduce the maximum thickness h_max_, by the following two steps:

Step 1- Evaluate the energy required to boil the heated metal to a depth determined by the pulse duration. For this we use equation (2) in which h_p_ is given by equation (1). This step provides us with the maximum energy E_max_ that the layer can accept which still can serve for jetting.

Step 2- Consists of the calculation of the maximal thickness that could be transferred with Emax . Recalling that the TIN-LIFT process depends on the melting front propagation up to free surface of the metal donor layer. To calculate the maximum layer thickness for which TIN-LIFT holds we first calculate the energy required to melt the entire layer thickness using equation (3). Since the lateral heat expansion is similar to the vertical thermal expansion, h_m_, the molten spot diameter will be D_L_+2h_m_ (see Eq. 4). Solving for these two steps numerically (using Matlab) we obtain the maximal donor thickness.

1. **Printing recipe:**

The jetting of a single metal droplet produces an opening in the metal donor layer surrounded by an extended heat-affected zone (HAZ). The HAZ determines the minimal distance to the next jetting spot position. The size of the opening and the HAZ range reflect the transient thermo-mechanical processes involved in the droplet formation and jetting. The HAZ must be taken into consideration when preparing a printing recipe in order to maintain high print quality but also to optimize the utilization of the printable metal layer. In the current case, where sub-nanosecond pulses and thin metal donors are used, a step size on the order of the laser spot size suffices to avoid HAZ effects from neighboring jetting sites. Specifically, for a 22 µm spots we have found that the minimal step should be D_HAZ_ = 30 µm.

The printing recipe consists of dividing the pattern into N square cells of area D_HAZ_^2^ each. With the donor held at a fixed position, we print first one droplet per each cell. Then we proceed and print droplets at distances dx=dy=D_HAZ_/K_cell_ , when K_cell_ is an integer (this is the case of equal drops distribution along the rows and columns). There is a total of K_cell_^2^ droplets per unit cell. This defines a single printed layer, the thickness of which is determined by the droplet volume and overlap. In order to build up thicker structures the same recipe is repeated several times.

1. **Adhesion:**

The adhesion of the printed copper droplet to metal oxides is typically poor, therefore a pre-treatment to remove the oxide is essential whenever we print on aluminum or copper foils (with their native oxides). On noble metals no adhesion problem was observed. On plastic, ceramic or epoxy (FR#4) we observe bouncing of the jetted droplet due to local heating at the contact point and the resulting pressure. In order to overcome this problem and provide good adhesion we had to pattern the surface prior to printing. This was typically done by laser ablation (otherwise, a mechanical roughening is an option) in order to generate controlled roughness on a scale similar to the droplet size. With this approach we managed to obtain good overall adhesion of printed metal structures on various polymeric substrates.
